# Decision-making of citizen scientists when recording species observations

**DOI:** 10.1038/s41598-022-15218-2

**Published:** 2022-06-30

**Authors:** Diana E. Bowler, Netra Bhandari, Lydia Repke, Christoph Beuthner, Corey T. Callaghan, David Eichenberg, Klaus Henle, Reinhard Klenke, Anett Richter, Florian Jansen, Helge Bruelheide, Aletta Bonn

**Affiliations:** 1 German Centre for Integrative Biodiversity Research (iDiv) Halle-Jena-Leipzig, Puschstraße 4, 04103 Leipzig, Germany; 2 Institute of Biodiversity, Friedrich Schiller University Jena, Dornburger Str. 159, 07743 Jena, Germany; 3 Department of Ecosystem Services, Helmholtz-Center for Environmental Research - UFZ, Permoserstraße 15, 04318 Leipzig, Germany; 4 Department of Survey Design and Methodology, GESIS - Leibniz Institute for the Social Sciences, P.O. Box 12 21 55, 68072 Mannheim, Germany; 5 Institute of Biology/Geobotany and Botanical Garden, Martin Luther University Halle-Wittenberg, Am Kirchtor 1, 06108 Halle, Germany; 6 Department of Conservation Biology & Social-Ecological Systems, Helmholtz-Center for Environmental Research - UFZ, Permoserstraße 15, 04318 Leipzig, Germany; 7 Thünen Institute of Biodiversity, Bundesallee 65, 38116 Braunschweig, Germany; 8 Faculty of Agricultural and Environmental Sciences, University of Rostock, Justus-von-Liebig-Weg 6, 18059 Rostock, Germany

**Keywords:** Biodiversity, Ecological modelling, Human behaviour

## Abstract

Citizen scientists play an increasingly important role in biodiversity monitoring. Most of the data, however, are unstructured—collected by diverse methods that are not documented with the data. Insufficient understanding of the data collection processes presents a major barrier to the use of citizen science data in biodiversity research. We developed a questionnaire to ask citizen scientists about their decision-making before, during and after collecting and reporting species observations, using Germany as a case study. We quantified the greatest sources of variability among respondents and assessed whether motivations and experience related to any aspect of data collection. Our questionnaire was answered by almost 900 people, with varying taxonomic foci and expertise. Respondents were most often motivated by improving species knowledge and supporting conservation, but there were no linkages between motivations and data collection methods. By contrast, variables related to experience and knowledge, such as membership of a natural history society, were linked with a greater propensity to conduct planned searches, during which typically all species were reported. Our findings have implications for how citizen science data are analysed in statistical models; highlight the importance of natural history societies and provide pointers to where citizen science projects might be further developed.

## Introduction

Citizen scientists—or volunteers contributing to scientific projects—increasingly take part in biodiversity monitoring by reporting species observations^1,2^. In Europe, for example, 87% of the participants in species monitoring are volunteers^3^. Chandler et al.^2^ estimated over half of the data in the Global Biodiversity Information Facility (GBIF)—the largest global biodiversity database—comes from citizen science platforms. Species observations can be submitted by volunteers to a growing number of global platforms, such as iNaturalist and eBird, or regional platforms such as iRecord in the UK. The quantity of data is only set to increase as citizen science expands across people, places and taxa^4^. Citizen science data are also increasingly used in scientific research to address a broad range of questions about the large-scale patterns and long-term changes of biodiversity^5–7^. However, the quality of the data within citizen science platforms has been questioned^8,9^, potentially leading to a barrier of their widespread use^10^. There is a pressing need to better understand the heterogeneity within citizen science data to ensure that they are appropriately used in biodiversity research as well as conservation policy and practise.

Citizen scientists make many decisions—before, during, and after observing species—that can affect different aspects of the data that they collect and report. In large part, individual variability in decisions is constrained by the level of structure of the project. Citizen science projects range from unstructured (i.e., little to no training needed and few to no protocols to follow; e.g., iNaturalist) to semistructured (i.e., few protocols but important metadata collected as part of the recording process; e.g., eBird) to structured (i.e., a priori temporal and spatial sampling designs, often with trained volunteers; e.g., Reef Life Survey)^11^. In structured citizen science projects, participants follow a common protocol that guides many of the data collection decisions. However, the majority of species occurrence data come from unstructured citizen science projects, probably because of trade-offs in citizen science project design—the more protocols, the less participation^11^. In unstructured citizen science projects, individual observers make many independent decisions about, for instance, the focal types of habitat and species, the sampling times and methods, and survey durations. Variation among individual observers in these decisions, thus, creates heterogeneity in the data^11–13^. Moreover, in platforms compiling unstructured data, species occurrence records are usually not accompanied by any information about sampling methods and decisions^14^. Analysis of unstructured citizen science data often has to make many assumptions about how the data were collected. Violation of the assumptions of commonly used statistical models for unstructured data, such as occupancy detection models, can lead to inaccurate and/or imprecise predictions of the spatial or temporal patterns of species distributions^15,16^.

Many previous studies to understand the data collection decisions of citizen scientists have taken a data-driven approach by analysing the patterns in the available data^4,14,17–22^. Most of these studies focused on the spatial patterns of the data, for example finding evidence for higher sampling effort near human settlements and roads^19^ as well as sometimes within protected areas^20^. Other studies have analysed observer-level patterns of species record submission to reveal the large variation among people in terms of recording intensity, taxonomic specialization, and preference towards rare species^12,14^. Data collected by citizen scientists have also been compared with those collected by professional surveyors to reveal differences in taxonomic identification skills and focus^23–25^. These studies indicate that common species are often underreported by citizen scientists, while rare species can be over-reported^15,26–29^.

An alternative approach to understanding citizen science data is by directly asking citizen scientists about their data collection activities. Interviews and questionnaires have been used to examine the motivations to participate in citizen science^30–34^, changes in conservation awareness and attitudes from participation^35–39^, and the types of people most likely to participate^32,34,40,41^. Questionnaires have also helped to understand some methodological aspects of data collection, including how citizen scientists interpret survey instructions^23^ and study designs that promote continued engagement^42,43^. However, to our knowledge, there has not yet been a questionnaire study focused on understanding the data collection decisions of citizen scientists.

We developed a questionnaire to ask citizen scientists about all aspects of their decision-making when contributing a species observation to a citizen science platform or database. Our questions related to species observation methods, species and site selection, sampling effort, species identification uncertainty, as well as motivations to collect data and experience. We targeted our questionnaire towards people in Germany who voluntarily and independently collect species observations for unstructured citizen science schemes. We disseminated our questionnaire broadly to reach people who varied in expertise and taxonomic focus. By characterizing the decision-making process of citizen scientists collecting biodiversity data, we aimed to: (1) quantify the greatest sources of variability among observers, which may need to be incorporated into biodiversity models of unstructured data^16^; (2) examine whether variables related to experience and motivation could be used as proxies for data collection variation^44^; and (3) identify potential metadata that describes some of the variation among people and could be more routinely collected by citizen science data platforms^45^.

## Methods

### Participant recruitment

The questionnaire was open from 15^th^ October to 30^th^ November 2020 and disseminated via multiple streams: personal contacts, natural history societies, German citizen science biodiversity platforms (Naturgucker/Naturwerke), Facebook groups and Twitter feeds of natural history groups. The questionnaire cover page explained the target audience and rationale for the questionnaire. The target audience was explained as people who voluntarily collect observations of plants or animals in their spare time, outside of a large-scale standardized monitoring program, and report these observations to an authority or organization. People of all levels of experience were encouraged to participate, from beginners to occasional collectors to experienced observers and experts. Respondents received no financial reward for participation.

### Participants

Our analysis was based on a convenience sample of 1,645 individuals who originally viewed the questionnaire. Out of these individuals, 15% did not answer any questions, and 20% did not get beyond question 3. Our final sample included 899 respondents who reached at least the penultimate page of the questionnaire—out of which only 12 did not complete the final page requesting demographic information. This participation rate is comparable to established thresholds for response rates in survey research^46^. The majority of the respondents identified as male (67%) and were aged 45–65 years old (Table 1; Fig. S1). Geographically, respondents were widely spread across our study region of Germany (Fig. S2).

**Table 1:** Profile of the respondents.

n = 899	Proportion (%) or median value (interquartile range)
Female	32%
Age (years)	55 (45–65)
Member of natural history society	42%
Number of years of experience	11 (4–30)

### Questionnaire description

We designed the questions in accordance with methodological standards for surveys in the social sciences^47,48^. The title page of the questionnaire explained the rationale of the study and that participation in the questionnaire was voluntary. The questionnaire was approved as anonymous and not collecting any personal data by the legal department of the Helmholtz-Zentrum für Umweltforschung GmbH - UFZ and performed in accordance with relevant guidelines and regulations. The questionnaire was piloted on a pre-selected group of target participants and revised following their feedback.

Broadly, the main questions of interest could be grouped into the following nine sections.

1. Experience: participants were asked questions on the number of years collecting data and frequency of data collection, and, in a later section, on membership of natural history societies, formal knowledge of biodiversity monitoring and participation in any large-scale structured monitoring schemes.
2. Motivations: participants were asked to rate the importance of ten different aspects about why they record biodiversity on a 5-point Likert scale ranging from 'not important at all' to 'very important'. Our selection of motivation factors was guided by similar ones included in other studies, including both intrinsic factors (motivated directly by enjoyment of the activity) and extrinsic factors (motivated for reasons outside of enjoyment of the activity itself)^49^. For instance, we included ‘have fun exploring’ as an intrinsic motivation and ‘support conservation’ as an extrinsic motivation.
3. Survey types: participants were asked to report what proportion of their species observations come from different species survey types: active and planned species surveys (i.e., going to a place with the intention of looking for species), opportunistic observations not seen during an active search or observations made using traps—on a 5-point Likert scale ranging from ‘none’ to ‘all’.
4. Active searches: participants were asked to rate the frequency with which they reported different kinds of species (e.g., all observed species or rare species only) on a 5-point Likert scale ranging from ‘never’ to ‘very often’ during an active and planned search and how long they typically spend looking for species (answering in minutes or hours).
5. Opportunistic observations: participants were asked to rate the frequency with which different scenarios (e.g., observations of rare species or simultaneous observations of many species) triggered opportunistic observations on a 5-point Likert scale ranging from ‘never’ to ‘very often’.
6. Trap use: if participants previously indicated that they used traps, they were asked to rate the frequency with which they reported different kinds of species collected in their traps on a 5-point Likert scale ranging from ‘never’ to ‘very often’ and how long the traps were left active (answering in hours or days).
7. Species ID uncertainty: participants were asked the frequency with which they dealt with uncertainty about the taxonomic identification in different ways (e.g., not report or guess), on a 5-point Likert scale ranging from ‘never’ to ‘very often’.
8. Locations: participants were asked to rate how often they looked for species in different habitats (e.g., forests, grasslands) on a 5-point Likert scale ranging from ‘never’ to ‘very often.’.
9. Consecutive surveys: participants were asked to rate how likely they were to report seeing a species again in the same place according to different time-periods since the previous detection of the same species, on a 5-point Likert scale ranging from ‘not at all likely’ to ‘very likely’.

We asked participants to focus their answers with reference to their main taxonomic group of interest (options given: birds, plants, butterflies/moths, beetles, bees, dragonflies, amphibians/reptiles or other, to be specified). This was to simplify interpretation of the results, since people may survey differently for different taxon groups. We also asked people about their demographics (age, gender, location at a coarse postcode level), but this section was left optional in case of concerns about personal identification.

We organized and disseminated the questionnaire using the LimeSurvey web application. We used data-piping to ensure that questions remained logical according to responses to previous questions and to remind people of their selected focal taxon group within the question phrasing. We randomized the order of items within a question (e.g., in the question about motivation factors) to avoid any order effects, unless there was a logical order to the items. We also used validation to reduce mistakes in data entry (e.g., only allowing integers of maximum two characters for questions about number of years of experience). Finally, we ensured questions were specific with respect to a time-frame when necessary and kept the time frame recent (Spring and Summer 2020) to assume reasonable levels of recall. The questionnaire is available in “Appendix A” (original in German) and “Appendix B” (translation in English) and a summary of the items for each main question group is in Table S1.

### Statistical analysis

Description: We conducted a descriptive analysis of the responses and visualized the numbers/proportions of people responding to each question with each answer option.

Linkages: We examined correlations among participant’s responses across all main questions. We employed either polychoric, polyserial or Pearson correlations for the relationships between two ordinal variables, one ordinal variable and one continuous variable or two continuous variables, respectively. We excluded ‘don’t know’ responses, which were consistently less than 5.5% of total responses for any item, and used the complete pair-wise data available for each correlation. We visualized the correlations using a chord diagram. Here, we were especially interested in the strength of the correlations between experience/motivations and data collection decisions.

Dimension reduction: We ran multiple analyses to assess whether responses to the questionnaire could be simplified to a smaller set of dimensions based on correlations among participants’ responses to each question. We excluded questions about traps from these analyses since they were only applicable to a small proportion of the participants. We used the subset of data from respondents who did not respond ‘don’t know’ to any question (n = 532 respondents). We first ran varimax-rotated principal components analysis (PCA) within each question group separately to identify the items that aligned most strongly with the first and second main axes of responses. Correlations among the top items of each question were then examined across question groups using varimax-rotated PCA and cluster analysis. For the cluster analysis, all values were scaled to between 0 and 1 and we used k-means partitioning, selecting the first three groups for simplicity of interpretation. All analyses and visualizations were done in R 4.1.0.

### Ethics declaration

For informed consent by the participants, the title page of the questionnaire explained the rationale and purpose of the study and that participation in the questionnaire was voluntary. The questionnaire was approved as anonymous and not collecting any personal data by the legal department of the Helmholtz-Zentrum für Umweltforschung GmbH - UFZ and performed in accordance with relevant guidelines and regulations.

## Results

### Experience and motivations

**Figure 1:**
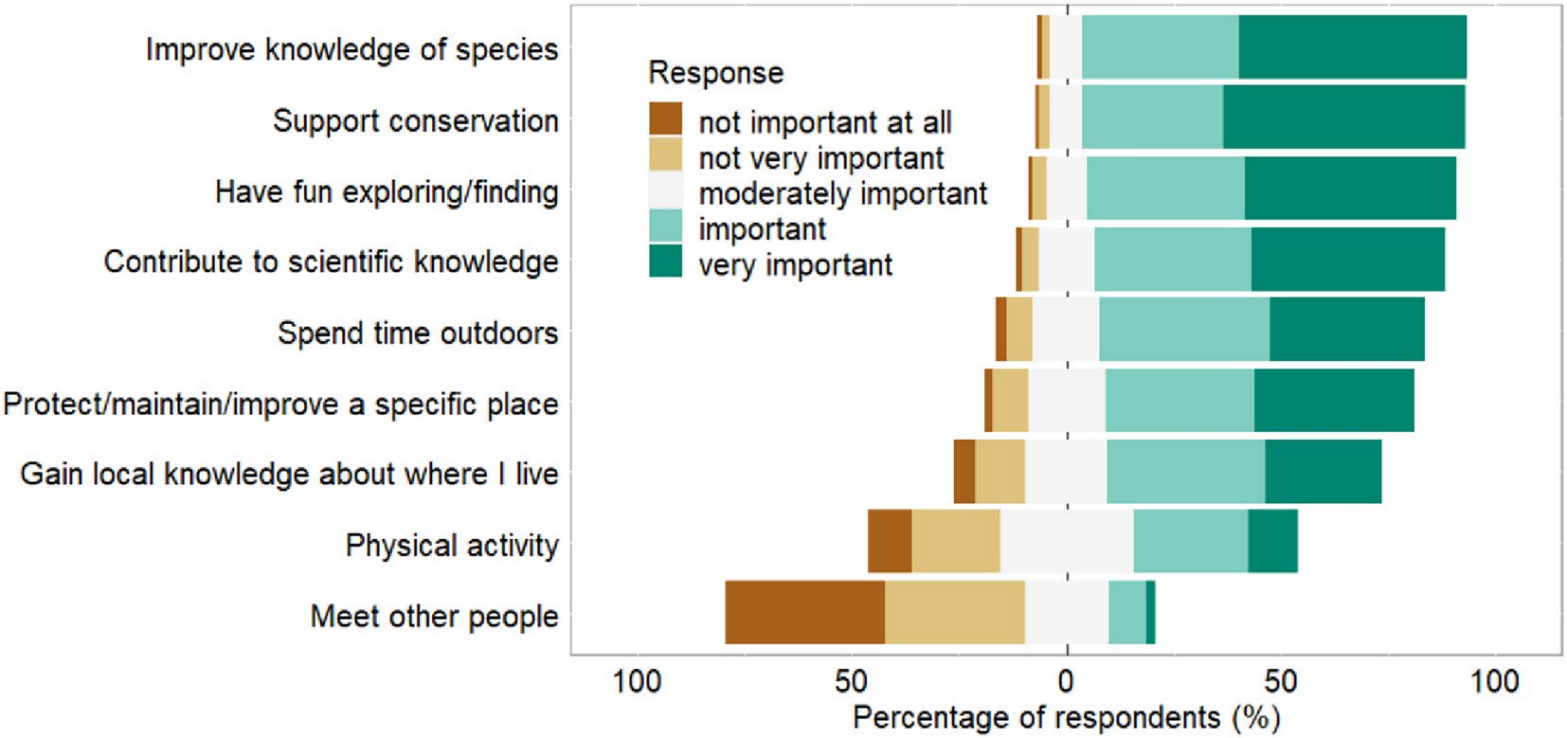
Motivations of respondents to collect species observation data. Respondents were asked to rate the importance of each item. Items are ordered in the plot by the % responding ‘important’ or ‘very important’.

Respondents ranged between beginners and those with more than 50 years of experience collecting species observation data, but the median number of years of experience was 11 years (Table 1; Fig. S3). Most respondents collected species data at least on a weekly basis during spring or summer 2020 (Fig. S3). The most important motivating factors to collect data were supporting conservation (very important for 56.4% of people) and improving knowledge of species (very important for 53.2% of people) (Fig. 1). In contrast, some of the least important reasons for data collection included ‘physical activity’ (very important for 11.4% of people) and ‘meeting other people’ (very important for 2.1% of people).

Since our survey questions related to activity during spring/summer 2020 when people might have been restricted due to COVID-19 lockdowns, we also asked whether observation/reporting of species was different this year compared to previous years. Most people (64%) said that they had been unaffected in terms of data collection activity. Similar proportions of people said they were more (13%) or less (14%) active in 2021 compared with previous years. For the remaining 8%, this year was their first year.

### Typical survey characteristics

- Taxonomic foci

The most common focal taxonomic groups were birds (41%) followed by plants (18%), different insect groups (butterflies/moths, beetles, dragonflies/damselflies and bees) (19%, collectively), and amphibians/reptiles (9.2%) (Fig. S4). Most people collected data on any species within their identified focal group (Fig. S4). However, there were specialists, especially within the most species-rich groups, for instance, 40% of all beetle data collectors focused on specific beetle subgroups such as ground beetles (Carabidae) (Fig. S4).

(b) Types of surveys

**Figure 2:**
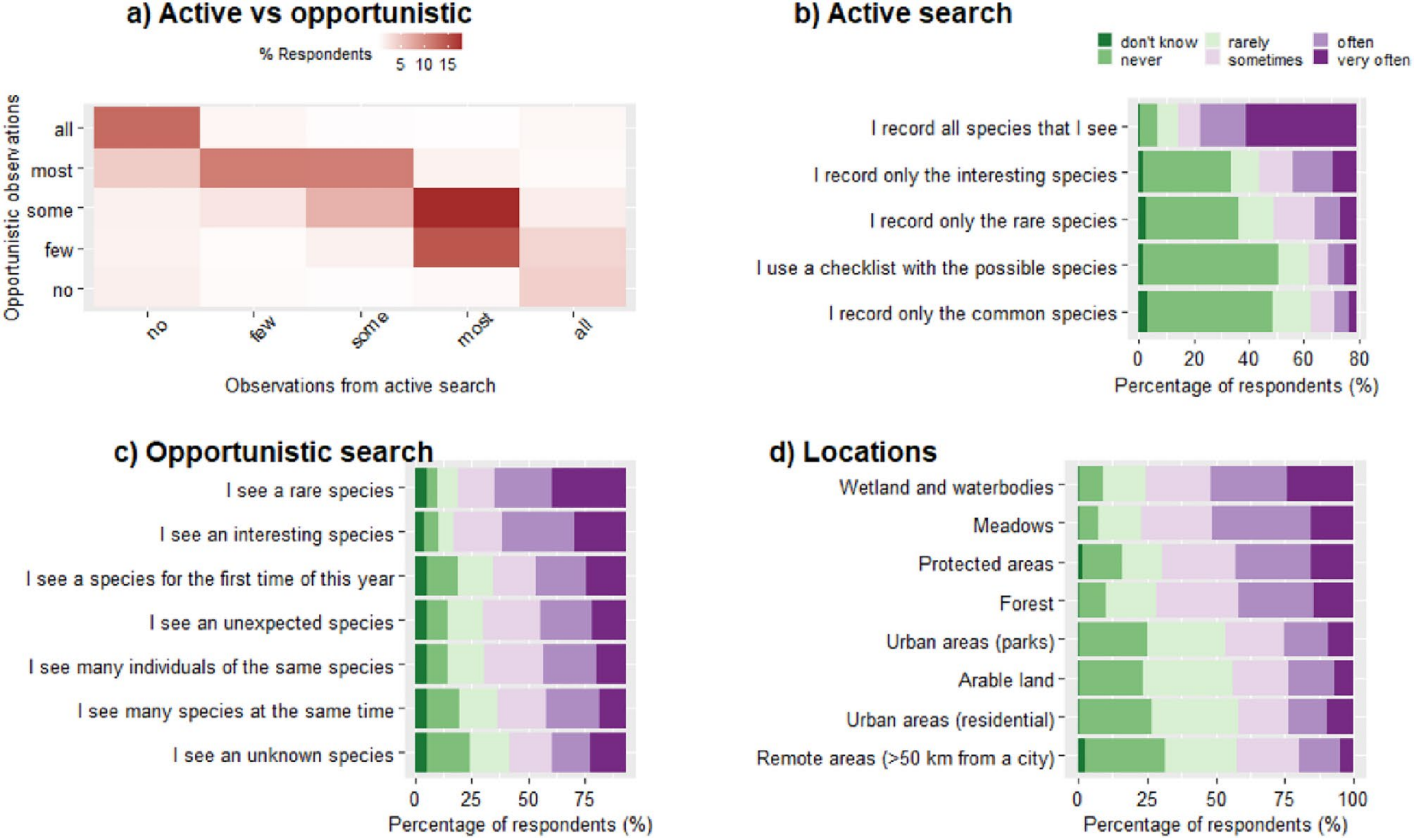
Survey patterns: (**a**) the proportion of species observation data that are made by an active/planned search compared with observations that were opportunistic; (**b**) species that are reported during an active/planned search; (**c**) triggers of an opportunistic observation; (**d**) locations/habitats in which people actively look for species.

In total, 45% of people reported that most or all of their species observations came from active/planned surveys; while 43% reported that most or all came from opportunistic observations. Typically, people reported to carry out a combination of active/planned surveys and opportunistic surveys (Fig. 2a). Traps were used by few recorders (< 5% of respondents) focused on specific taxonomic groups; for instance, butterfly/moth specialists used different kinds of light traps (Table S2 for range of traps in use).

During an active/planned search, people stated that they reported most (38.5% of respondents) or all (14%) of the species that they saw (Fig. 2b). Consistent with this, few people reported only rare, only common or only species deemed interesting during active searches. Surveys were usually 2–3 h long, with some variation among taxonomic groups (Fig. S5).

(c) Triggers of opportunistic observations

When a species was reported after an incidental observation, observation of a rare species was the most common trigger (often or very often for over 50% of people). The least common triggers of opportunistic observations were related to species abundance and species richness, i.e., seeing many individuals of a species or seeing multiple species at the same time (Fig. 2c).

(d) Preferred locations

Respondents reported visiting different types of habitats to look for species, but with a tendency to more often visit semi-natural habitats than more human-modified habitats within urban or arable land (Fig. 2d). The most preferred places were open habitats such as meadows and wetlands/water bodies, which were visited often or very often by 52% of people. The least preferred habitats were arable land and urban areas (especially non-green), which were visited often or very often by 24% of people.

(e) Dealing with species identification uncertainty

When there was some uncertainty about species identification, most people used an identification guide to help (often or very often for over 84%). The least common approach was to simply guess the species identification (Fig. S6).

(f) Consecutive surveys

The reporting of a species observation on a given day depended on whether the person had previously reported the same species in the same location (Fig. S7). Most people were unlikely to record the same species twice on the same day in the same place, but were increasingly likely to report it again with increasing time since the last observation: for instance, 25.2% were likely to report it again if seen in the previous week while 44% would report it again if seen in the previous year (Fig. S7).

### Linkages across questions groups

**Figure 3:**
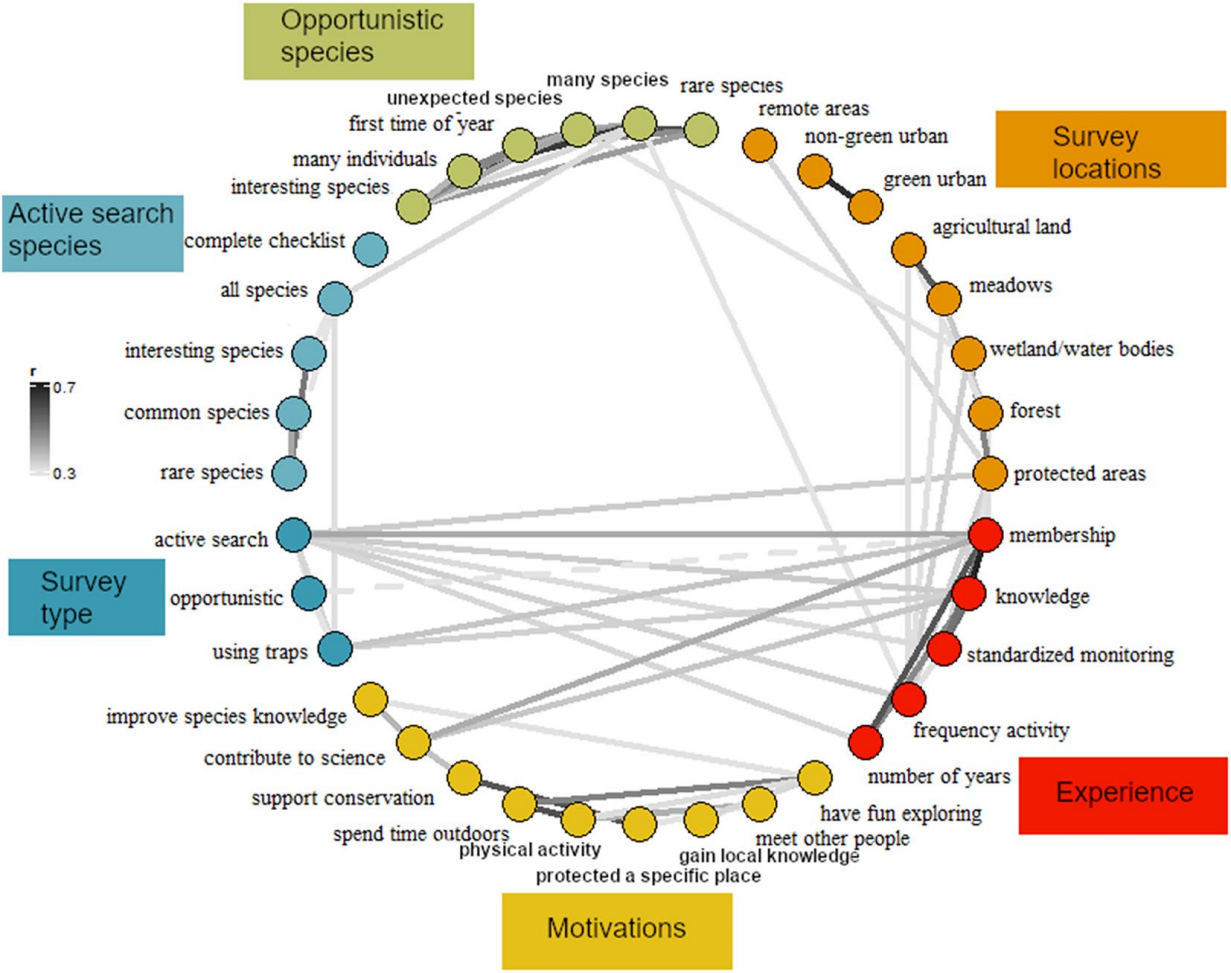
Plot showing the correlations among responses across all questions. Lines connecting two items indicate a correlation with absolute strength of 0.3 or greater between answers of each item, with darker shading indicating stronger correlation strengths. Negative correlations (very few) are shown by a dashed line. Table S1 gives a fuller description of all question items.

Motivations to report observations were not linked with any data collection methods or preferences, but there were some linkages between motivations and experience, albeit weak ones (Fig. 3). Contributing to science was an especially motivating factor for members of a natural history society (r = 0.4) and those who regard themselves as having knowledge of biodiversity monitoring (r = 0.35) (Fig. 3). Experience was also linked with some aspects of data collection, especially survey types and locations (Fig. 3). For instance, active/planned surveys were linked with experience attributes, especially membership of a natural history society (r = 0.41). Active/planned searches were also linked with visiting protected areas (r = 0.34). The proportion of records coming from active searchers was negatively correlated with the proportion coming from opportunistic observations (r = − 0.7), which is consistent with these search types being understood as mutually exclusive by the respondents. Frequency of activity was also linked with visiting multiple habitat types, including agricultural land.

### Dimension reduction to identify main axes of variation

**Figure 4:**
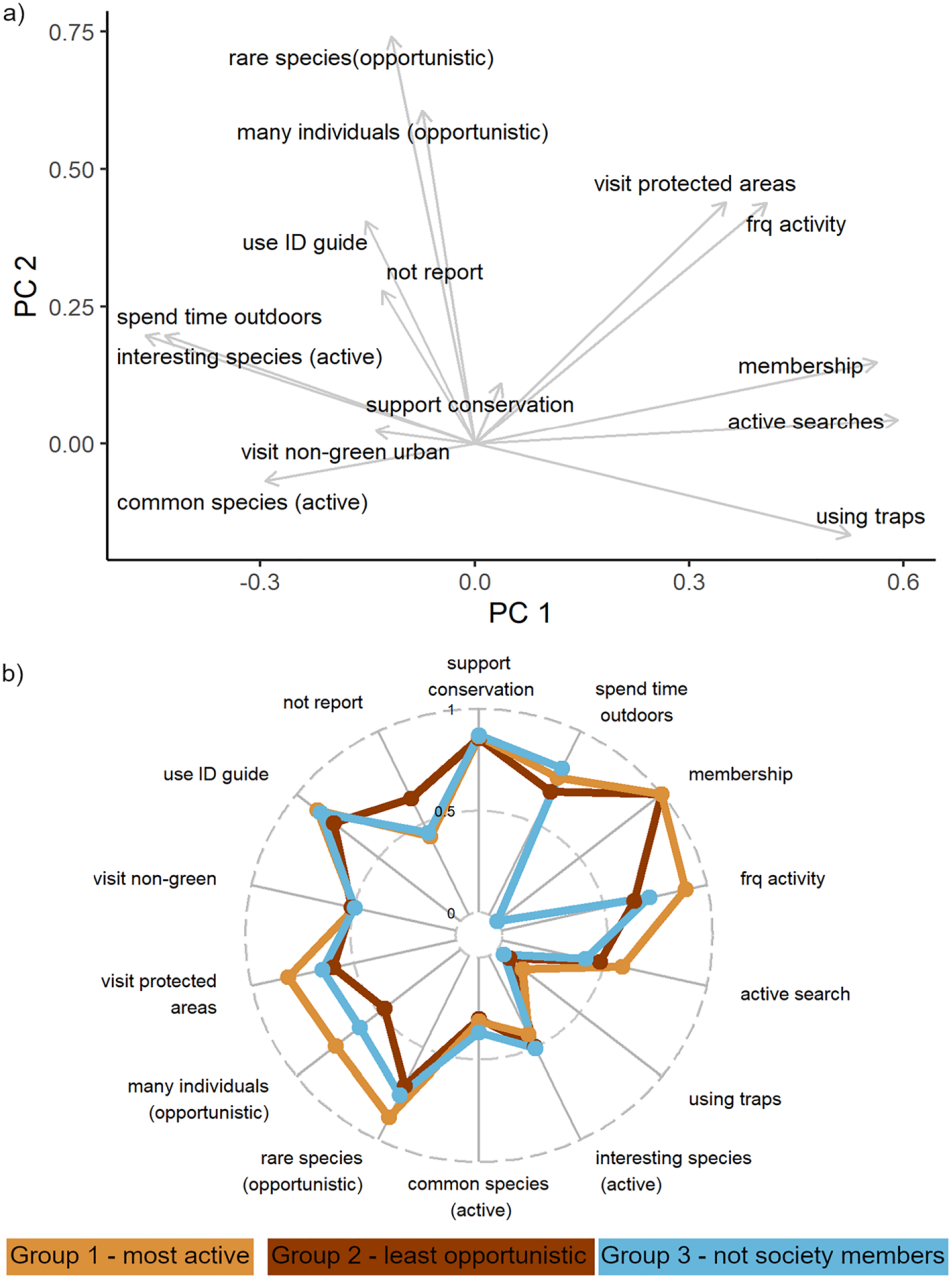
Dimension reduction: (**a**) a PCA analysis of the top items of all question groups (PC axis 1 explained 13% of the variation and PC axis 2 explained 12% of the variation) and (**b**) characteristics of the main respondent groups from a k-means cluster analysis. Points on each axis represent the mean value for people within each group (separated by different colours) scaled between the minimum and maximum values. Table S1 gives a fuller description of all question items.

A PCA of responses to the top items (Table 2; Fig. S8) within all questions revealed that the first two components explained 25% of the variation among respondents. Natural history society membership and active/planned searches loaded more strongly on the first component (Fig. 4a). The second component was most associated with rare species as a trigger of opportunistic observations (Fig. 4a).

**Table 2:** Items explaining variation among people, as assessed by which items loaded most strongly onto each of the first two principal components of a PCA.

Question group	Key items associated with variation among respondents
Experience	Society membership versus frequency of activity
Motivations	Spend time outdoors versus support conservation
Survey type	Active/planned search versus using traps
Active search species	Interesting species versus common species
Opportunistic species triggers	Rare species versus many individuals at the same time
Survey locations	Protected areas versus non-green urban areas
Species ID uncertainty	Use an identification guide versus not report

Figure S8 shows the PCA biplots for each question group and Table S1 lists all items within each question group.

The best-defined three groups of respondents from a cluster analysis varied mostly in natural history society membership, propensity to conduct active searches, frequency of activity and triggers of opportunistic searches (Fig. 4b). Group 1 were the most active (in terms of frequency of activity) and were members of natural history societies and the most likely to conduct active searches. Group 2 were also consistently members of natural history societies but the least likely to report species opportunistically, and most likely not to report an observation when there was some uncertainty about species identification. Group 3 were not members of natural history societies and the least likely to conduct active searches. Groups were generally similar in terms of being motivated by supporting conservation.

## Discussion

Our study of citizen scientists across Germany demonstrates the diversity of decision-making and sampling methods underlying species occurrence records, consistent with previous studies analysing patterns in the available species data^12,14^. Nonetheless, by directly asking citizen scientists, our questionnaire approach allowed us to uncover some new patterns in the recording process of species observations. We found that: (1) volunteers were most often motivated by improving species knowledge and supporting conservation but motivations were not linked to data collection methods; (2) people often report observations from both opportunistic and active/planned surveys; however, the likelihood of complete surveys (i.e., reporting all species within their main focal taxon group) was highest during active/planned searches; (3) active/planned search were more typical of members of a natural history society; and (4) there was some temporal dependence in recording behaviour among consecutive surveys. Collectively, our findings have implications for how citizen science data are used in biodiversity research and highlight where citizen science projects might be further developed.

### Implications for using citizen science data in biodiversity research

While various statistical methods have been proposed to account for the heterogeneity of unstructured citizen science data^16,50,51^, few studies have aimed to understand the data by directly asking citizen scientists about their data collection methods. In the absence of metadata on sampling methods, current approaches to model these data often involve creating proxies of survey type, effort and observer skill based on the available information^50,51^. For instance, many studies have calculated list length—the number of species reported on a given visit—and used this as a covariate to account for variation in survey type and effort^51^, assuming that recorders submitting more species were more likely to be performing a complete and/or longer survey. Similarly, other studies used proxies of observer experience, based on the total number of submitted records, to model variation in the data collected by different people^44^.

Our findings lend some support to these approaches but also reveal some further nuances. First, our questionnaire supported a contrast in the data coming from different survey types—specifically opportunistic observations compared with observations from active and planned searches. We found that the same people report observations from both opportunistic surveys and from active/planned surveys. We also found that people acted differently in each search type—reporting all species during active/planned searches but often only rare species opportunistically. Since rare species rather than groups of species at the same time were more typical triggers of opportunistic observations, a list length covariate may explain some, but not all, of the variation in survey types. Second, we found that experience-related variables were also linked with propensity to conduct active/planned searches, also supporting attempts to include experience proxies in analysis of unstructured data^44^.

Our data also revealed often overlooked patterns of data collection behaviour that have implications for analysis of citizen science data. For instance, we found that there was temporal dependence in recording behaviour. Specifically, people were less likely to report an observation of a species if they had already reported the species in the same place at previous time points. Methods to deal with such patterns have been suggested already, for instance, by borrowing methods developed to correct for trap-happiness found in some mark-recapture studies^16^, but they have rarely been applied in analyses of unstructured citizen science data so far. Studies of human preference decisions have tested the effects of novelty and familiarity^52,53^. These studies provide some support for a novelty preference within a nature setting, but at the same time highlight that this effect is context-dependent, for instance, depending on what task is being performed. However, such a novelty preference could explain the reduced likelihood of re-reporting a species at the same place as well as the increased likelihood to report a rare species opportunistically.

Since variation among people could not be reduced to just a few components, our findings are consistent with previous studies showing that people vary along multiple axes of recording behaviours^12^. Statistical methods might be able to account for some of this unmeasured heterogeneity using mixed-effects models with different random effects, e.g., for site and observer^16^, but some variation is likely to go unexplained since we show that observers themselves vary in their behaviour on different surveys. Hence, alongside developments in statistical methods, there should be better recognition of the importance of more detailed survey-level metadata to make the best use of unstructured citizen science data^45^.

### Increasing the value of data by more detailed metadata

Lack of metadata associated with species occurrence records can lead to data heterogeneity being equated with low data quality. Hence, more detailed metadata could increase the utility of unstructured data for biodiversity research^11^. Some aspects of data collection can be characterized post hoc (e.g., survey habitat might be extractable using supplied geographic coordinates). Other metadata (e.g., time of day or survey duration) might be automatically identified by some software applications. However, other metadata needs to be provided manually by citizen science participants. Some platforms, such as eBird, already request that data submitters declare which kind of bird survey is associated with a set of observations, e.g., a complete checklist survey in which all observed species were reported^45^. Our analysis reveals the challenges of defining complete checklists for a broader range of taxa because complete checklists can only be defined with respect to a specific target taxonomic group. Some insect groups such as butterflies and dragonflies can represent target taxonomic groups since we found that few people specialized within these groups. By contrast, for other insect groups, especially species-rich groups such as beetles, the target taxonomic groups might be better defined at lower taxonomic levels, since here we found a larger proportion of specialists. Hence, metadata would need to be collected on both survey type (complete checklist or not) as well as target taxonomic group of interest.

### Implication for citizen science project design

We found that people were motivated by both intrinsic and extrinsic factors^49^, but the top rated motivation factors were both extrinsic, suggesting that people are especially motivated by perceived benefits beyond enjoyment of the activity itself, especially the opportunity to increase species knowledge and support conservation^54,55^. To nurture long-term participation in citizen science, mechanisms could be put into place that provide feedback to participants on how their data are contributing to biodiversity research and conservation. Such mechanisms could lead to higher levels of long-term participation and retention in citizen science projects^54,56^. Citizen science participation has been linked with changes in attitudes towards conservation and conservation behaviours^57^). Since we found that motivations covaried somewhat with experience-related attributes, including membership of a natural history society, different projects and practices might encourage the involvement of participants with different backgrounds. Motivations did not consistently correlate with any data collection behaviour, hence, different types of citizen science projects can contribute useful data on species occurrence^58^.

Citizen science data is arguably most useful for ecological research by providing large spatial and temporal data sets that cannot be accomplished by academic scientists alone. Our analysis indicated that people most commonly look for species in open habitats, such as wetlands, water bodies and meadows^13^, and less often look in urban and agricultural areas. Despite being potentially less preferred, urban areas still are hotspots of data collection because of the large number of people living nearby^19^. However, agricultural areas, which are typically underrepresented in species occurrence record databases, and also less preferred as places to look for species according to our study, might need to be targeted by dedicated citizen science projects or standardized scientific surveys. Recent studies have developed methods to highlight under-sampled areas in order to encourage data collection and balance sampling effort across the landscape^59^.

### Key role of natural history societies

We found that members of natural history societies had a greater propensity to carry out active/planned searches, which were associated with comprehensive surveys of all species, suggesting that their data might be especially useful for biodiversity research. Other studies have already shown the value of unstructured data from natural history groups for assessment of large-scale and long-term trends of insect taxa, which are mostly not targeted by large-scale structured monitoring schemes^60,61^. Natural history societies may thus serve as both an important mechanism for generating valuable biodiversity data, but also as a mechanism for recruitment of volunteers to contribute data and sharing of taxonomic knowledge and expertise. Natural history societies could be better supported in their needs for this role by both governmental agencies as well as research funders and invited to collaborate with professional scientists.

### Study limitations and caveats

Although our study was able to reveal multiple aspects of citizen scientist decision-making, it had some particular aspects that might affect the generality of our findings. First, it is likely that due to the nature of our survey and the snowballing approach of dissemination, many of the respondents were probably the most active citizen scientists at the high end of recording frequency. Hence, our findings may not reveal the full breadth of recording patterns across citizen scientists. However, since most data are collected by a small number of highly active recorders^12–14^, our findings remain highly useful to understand recording activities underlying the data within big databases that are commonly used for ecological research. Second, we focused on the year 2020 to ascertain current behaviour, but recording activities might have been affected by the COVID-19 pandemic. As elicited by a specific question related to this, few people reported to have been affected; although, probably the most affected people did not participate in our questionnaire at all.

## Conclusions

Harnessing the full potential of citizen science data requires better understanding of how the data are collected. We demonstrated the heterogeneous nature of individual variability in citizen scientists, and their decisions as to when, how, and where to sample biodiversity. Improved metadata associated with species observations can help devise the most appropriate analysis for questions on biodiversity change. Studies of citizen scientists, such as ours, as well as studies of patterns in the available data^12^ will help paint a better picture for how to extract the maximum information for biodiversity research.

## Supplementary Information

Supplementary Information 1.

Supplementary Information 2.

Supplementary Information 3.

## Data Availability

Data are published in the iDiv data repository, https://idata.idiv.de  (ID: 3512). Code to analyse the data is available at: https://github.com/bowlerbear/Citizen_science_manuscript.
