# Supplementary material for: Decision-making of citizen scientists when recording species observations
Source: Sci Rep. 2022 Jun 30;12:11069. doi: 10.1038/s41598-022-15218-2 (PMC9245884; doi:10.1038/s41598-022-15218-2)
Supplement: Supplementary file 1 — Supplementary Information 1. [file 41598_2022_15218_MOESM1_ESM.docx]

**sMon Beobachtungsdaten**

**Fragebogen zur Erfassung von Beobachtungsdaten**

Liebe Naturbeobachterinnen und Naturbeobachter,

wir brauchen Ihre Hilfe.

Ihr Engagement bei der Meldung von Artenbeobachtungen ist sehr wichtig für die Biodiversitätsforschung. Diese Umfrage dient dazu, die verschiedenen Erfassungsmethoden von Naturbeobachter*innen in Deutschland zu besser zu verstehen. Ihre Erfahrung wird uns helfen, bessere Auswertungsmöglichkeiten für Biodiversitätstrends zu entwickeln. Wir interessieren uns dafür, wie Menschen Entscheidungen treffen, wenn sie ihre Beobachtungen sammeln und wie sie diese dokumentieren. Ein Großteil solcher Beobachtungen/Erfassungen findet nicht im Rahmen strukturierter Monitoringprogramme statt (z.B. Tagfalter-Monitoring Deutschland). Vielmehr werden die meisten Beobachtungsdaten von einer Vielzahl von Beobachter*innen nach unterschiedlichen Kriterien erfasst und z.B. über Apps übermittelt oder in Online-Portalen eingetragen (z.B. ornitho.de, artenfinder.rlp). In dieser Umfrage zielen wir auf eben solche Beobachtungen und deren Methoden ab.

**Wer führt die Umfrage durch?**

Wir sind Wissenschaftler*innen des Deutschen Zentrums für integrative Biodiversitätsforschung (iDiv) Halle-Jena-leipzig und führen die Umfrage im Rahmen des Projekts sMon- Biodiversitätstrends in Deutschland (idiv.de/smon) durch. In sMon arbeiten wir eng mit Fachgesellschaften, Naturschutzverbänden bzw. -behörden und Forschungseinrichtungen in Deutschland zusammen, um Biodiversitätsdaten auszuwerten und um Trends zu ermitteln.

**An wen richtet sich die Umfrage?**

Die Umfrage richtet sich an Personen, die auf freiwilliger Basis in ihrer Freizeit Beobachtungen von Pflanzen oder Tieren sammeln und diese Beobachtungen einer Behörde oder Organisation melden. Menschen aller Erfahrungsstufen werden zur Teilnahme ermutigt, von Anfängern über gelegentliche Sammler bis hin zu sehr erfahrenen Beobachter*innen und Expert*innen. Beim Beantworten der Umfrage gibt es keine richtigen oder falschen Antworten.

**Hinweise zum Datenschutz**

Die Teilnahme an der Umfrage ist freiwillig. Die Erhebung und Verarbeitung der in dieser Umfrage erhobenen Daten erfolgt nach Datenschutz-Grundverordnung (EU-DSGVO). Die Daten werden nur für wissenschaftliche Zwecke verwendet; eine Weitergabe an Dritte erfolgt nicht. Alle Antworten und Angaben werden anonymisiert.

**Das Ausfüllen dieser Umfrage dauert etwa 15 Minuten.**

Sollten Sie weitere Fragen zum Inhalt oder Vorgehen bei der Umfrage haben, zögern Sie nicht, mich zu kontaktieren. Am Ende der Umfrage ist auch Platz für Kommentare.

Vielen Dank im Voraus für Ihre Teilnahme an dieser Umfrage.

Dr. Diana Bowler

Deutsches Zentrum für Integrative Biodiversitätsforschung (iDiv) Halle-Jena-Leipzig

Puschstr. 4

04103 Leipzig

E-Mail: [smon@idiv.de](mailto:smon@idiv.de); Web: www.idiv.de/de/smon.html

**Ihre Erfahrungen**

**Wie viele Jahre sind Sie schon in der Erfassung der Artenbeobachtungsdaten aktiv?**

In diesem Feld darf nur ein ganzzahliger Wert eingetragen werden.

Bitte geben Sie Ihre Antwort hier ein:

- Jahr/Jahre

**Wie oft haben Sie im Frühling oder Sommer 2020 Artdaten gesammelt?**

Bitte wählen Sie nur eine der folgenden Antworten aus:

- Täglich / fast täglich
- Wöchentlich / fast wöchentlich
- all zwei Woche/ fast all zwei Woche
- Monatlich / fast monatlich
- alle zwei Monate / fast alle zwei Monate
- Seltener

**Wie wichtig waren Ihnen die folgenden Aspekte, als Sie Beobachtungsdaten erfasst haben?**

Bitte wählen Sie die zutreffende Antwort für jeden Punkt aus:

|  | überhaupt nicht wichtig | wenig wichtig | mäßig wichtig | wichtig | sehr wichtig |
| --- | --- | --- | --- | --- | --- |
| Kenntnisstand über Arten verbessern |  |  |  |  |  |
| Zu wissenschaftlichen Erkenntnissen beitragen |  |  |  |  |  |
| Naturschutz unterstützen |  |  |  |  |  |
| Zeit im Freien verbringen |  |  |  |  |  |
| Körperliche Aktivität |  |  |  |  |  |
| Schutz/Erhalt/Verbesserung eines bestimmten Ortes |  |  |  |  |  |
| lokale Kenntnisse über mein Wohnumfeld gewinnen |  |  |  |  |  |
| andere Menschen treffen |  |  |  |  |  |
| Spaß beim Entdecken/Finden |  |  |  |  |  |

**Welche taxonomische Gruppe beobachten und melden Sie am häufigsten?**

**Bitte wählen Sie EINE Artengruppe.**

Bitte wählen Sie nur eine der folgenden Antworten aus:

- Pflanzen
- Käfer
- Libellen
- Schmetterlinge
- Bienen
- Amphibien/Reptilien
- Vögel
- Andere (Bitte spezifizieren Sie)

**Erfassen Sie vorrangig Beobachtungsdaten über eine bestimmte Untergruppe (z.B. eine Familie) innerhalb dieser Artengruppe?**

Bitte wählen Sie nur eine der folgenden Antworten aus:

- Ja, ich erfasse vorrangig Daten über eine bestimmte Untergruppe.
- Nein, ich erfasse nicht vorrangig Daten über eine bestimmte Untergruppe.
- Ich weiß nicht.

**Falls Ja - Bitte spezifizieren Sie die Untergruppe, über die Sie vorrangig Beobachtungsdaten erfassen.**

Bitte geben Sie Ihre Antwort hier ein:

**Bitte beantworten Sie die verbleibenden Fragen in der Umfrage in Hinsicht auf diese Artengruppe**

**Welche Plattform oder Plattformen nutzen Sie hauptsächlich zur Einreichung Ihrer Bienen -Beobachtungen?**

Bitte wählen Sie alle zutreffenden Antworten aus:

- naturgucker
- iNaturalist
- Artenfinder
- Naturblick
- Ich benutze keine Plattform
- Andere (Bitte spezifizieren Sie) :

**War Ihre Artenbeobachtung oder -berichterstattung im Frühling/Sommer 2020 aufgrund der Corona-Situation anders als in den Vorjahren?**

Bitte wählen Sie nur eine der folgenden Antworten aus:

- Nein, es war mein erstes Jahr.
- Nein, ich bin weitgehend genauso vorgegangen wie in den anderen Jahren.
- Ja, ich war weniger aktiv als in den anderen Jahren.
- Ja, ich war aktiver als in den anderen Jahren.

**In den nächsten Fragen interessieren wir uns dafür, wie Sie Artenbeobachtungen erfassen.**

**Wie viele der Bienen-Beobachtungen, die Sie im Frühling und Sommer 2020 gemeldet haben, waren:**

Bitte wählen Sie die zutreffende Antwort für jeden Punkt aus:

|  | keine | wenige | einige | die meisten | alle |
| --- | --- | --- | --- | --- | --- |
| das Resultat einer aktiven Suche (Sie sind zum Beispiel an einen bestimmten Ort gefahren, um gezielt nach Arten zu suchen) |  |  |  |  |  |
| oder zufällige Beobachtungen (ohne aktive Suche) |  |  |  |  |  |
| oder aus aufgestellten Fallen |  |  |  |  |  |

**Aktive Suche**

**Wenn Sie aktiv auf die Suche nach Bienen gegangen sind, wie lange suchten Sie typischerweise?**

Bitte geben Sie Ihre Antwort(en) hier ein:

- in Stunden
- oder in Minuten

**Wenn Sie aktiv auf die Suche nach Bienen gegangen sind, wie sind Sie bei der Sammlung von Beobachtungen vorgegangen?**

Bitte wählen Sie die zutreffende Antwort für jeden Punkt aus:

|  | weiß nicht | nie | selten | manchmal | oft | sehr oft |
| --- | --- | --- | --- | --- | --- | --- |
| Ich habe eine Checkliste mit den zu erwartenden Arten und sehe die Liste durch. |  |  |  |  |  |  |
| Ich erfasse alle Arten, die ich gesehen habe. |  |  |  |  |  |  |
| Ich erfasse nur die Arten, die ich interessant finde. |  |  |  |  |  |  |
| Ich erfasse nur häufige Arten. |  |  |  |  |  |  |
| Ich erfasse nur seltene Arten. |  |  |  |  |  |  |

**zufällige Beobachtungen**

**Was veranlasst Sie dazu, eine Bienen-Beobachtung zu melden, wenn Sie eine Art zufällig sehen (ohne aktive Suche)? Bitte geben Sie an, wie oft die folgenden Gründe bei Ihnen zur Meldung einer Beobachtung führen.**

Bitte wählen Sie die zutreffende Antwort für jeden Punkt aus:

|  | weiß nicht | nie | selten | manchmal | oft | sehr oft |
| --- | --- | --- | --- | --- | --- | --- |
| Ich sehe eine seltene Art. |  |  |  |  |  |  |
| Ich sehe viele Individuen der gleichen Art. |  |  |  |  |  |  |
| Ich sehe viele Arten zur gleichen Zeit. |  |  |  |  |  |  |
| Ich sehe die Art an einem Ort, an dem ich sie nicht erwartet habe. |  |  |  |  |  |  |
| Ich sehe die Art zum ersten Mal in diesem Jahr. |  |  |  |  |  |  |
| Ich sehe eine Art, die ich nicht kenne. |  |  |  |  |  |  |
| Ich sehe eine Art, die ich interessant finde. |  |  |  |  |  |  |

**Aufgestellte Fallen**

**Wenn Sie eine Bienen-Falle verwendet haben,wie lang war typischerweise das Erfassungs-Zeitfenster?**

Bitte geben Sie Ihre Antwort(en) hier ein:

- in Tagen
- oder in Stunden

**Welche Typen von Bienen-Fallen verwenden Sie? (Bitte trennen Sie diese jeweils mit einem Komma)**

Bitte geben Sie Ihre Antwort hier ein:

**Nach welchem Schema erfassen Sie die mit der Bienen-Falle gefangenen Arten?**

Bitte wählen Sie die zutreffende Antwort für jeden Punkt aus:

|  | weiß nicht | nie | selten | manchmal | oft | sehr oft |
| --- | --- | --- | --- | --- | --- | --- |
| Ich erfasse alle Arten. |  |  |  |  |  |  |
| Ich erfasse nur die Arten, die ich interessant finde. |  |  |  |  |  |  |
| Ich erfasse nur häufige Arten. |  |  |  |  |  |  |
| Ich erfasse nur seltene Arten. |  |  |  |  |  |  |
| Ich erfasse nur Arten, die noch nicht in meiner Sammlung sind. |  |  |  |  |  |  |

**Artenbestimmung**

**Was tun Sie, wenn Sie sich bei der Bestimmung einer Bienen- Art unsicher sind? Bitte geben Sie an, wie häufig Sie in diesem Fall folgende Dinge tun:**

Bitte wählen Sie die zutreffende Antwort für jeden Punkt aus:

|  | weiß nicht | nie | selten | manchmal | oft | sehr oft |
| --- | --- | --- | --- | --- | --- | --- |
| Ich vermute, um welche Art es sich handelt. |  |  |  |  |  |  |
| Ich melde die Art nicht. |  |  |  |  |  |  |
| Ich melde die Beobachtung auf der taxonomischen Ebene, die ich sicher identifizieren kann (z. B. Gattung oder Familie). |  |  |  |  |  |  |
| Ich bitte eine andere Person, meine Bestimmung zu überprüfen. |  |  |  |  |  |  |
| Ich nutze Hilfsmittel zur Bestimmung (z. B. das Internet oder ein Bestimmungsbuch). |  |  |  |  |  |  |

**Orte der Artenbeobachtung**

**Wenn Sie an den Frühling oder Sommer 2020 denken, wie oft haben Sie an den folgenden Orten nach Arten gesucht?**

Bitte wählen Sie die zutreffende Antwort für jeden Punkt aus:

|  | weiß nicht | nie | selten | manchmal | oft | sehr oft |
| --- | --- | --- | --- | --- | --- | --- |
| Schutzgebiete |  |  |  |  |  |  |
| Wald |  |  |  |  |  |  |
| Feuchtgebiete und Gewässer |  |  |  |  |  |  |
| Wiesen |  |  |  |  |  |  |
| Ackerland |  |  |  |  |  |  |
| Städtische Gebiete – Grünflächen (z.B. Parks) |  |  |  |  |  |  |
| Städtische Gebiete – bebaute Flächen (z.B. Häuser, Gehsteige, Straße) |  |  |  |  |  |  |
| Abgelegene Gebiete (z. B. mehr als 50 km von einer Stadt entfernt) |  |  |  |  |  |  |

**Stellen Sie sich vor, Sie besuchen einen Ort, den Sie schon einmal besucht haben. Dort sehen Sie eine Art, die Sie bei Ihrem letzten Besuch bereits identifiziert und gemeldet haben.**

**Wie wahrscheinlich ist es, dass Sie diese neue Beobachtung melden würden, wenn die letzte Beobachtung...?**

Bitte wählen Sie die zutreffende Antwort für jeden Punkt aus:

|  | weiß nicht | überhaupt nicht wahrscheinlich | wenig wahrscheinlich | mäßig wahrscheinlich | ziemlich wahrscheinlich | sehr wahrscheinlich |
| --- | --- | --- | --- | --- | --- | --- |
| … am selben Tag war. |  |  |  |  |  |  |
| … in derselben Woche war. |  |  |  |  |  |  |
| … im selben Monat war. |  |  |  |  |  |  |
| … im selben Jahr war. |  |  |  |  |  |  |
| …in einem früheren Jahr war. |  |  |  |  |  |  |

**Persönliche Angaben**

Zuletzt würden wir gerne noch Informationen zu Ihrer Person aufnehmen. Sie bleiben dabei anonym - aus den persönlichen Angaben kann nicht auf die Identität einzelner Teilnehmender der Umfrage geschlossen werden. Wenn Sie keine persönlichen Angaben machen möchten, klicken Sie unten auf „Absenden".

**Ich bin**

Bitte wählen Sie nur eine der folgenden Antworten aus:

- weiblich
- männlich
- divers

**Zu welcher Altersklasse gehören Sie?**

Bitte wählen Sie nur eine der folgenden Antworten aus:

- 19 oder jünger
- 20-29
- 30-39
- 40-49
- 50-59
- 60-69
- 70-79
- 80+

**Nehmen Sie an einem groß angelegten standardisierten Monitoringsystem teil (z. B. Tagfalter Monitoring Deutschland)?**

Bitte wählen Sie nur eine der folgenden Antworten aus:

- Ja
- Nein

**Besitzen Sie Fachkenntnisse im Bereich des Biodiversitätsmonitorings?**

Bitte wählen Sie nur eine der folgenden Antworten aus:

- Ja
- Nein

**Wo haben Sie die Fachkenntnisse hauptsächlich erworben?**

Bitte wählen Sie nur eine der folgenden Antworten aus:

- Universität/ Hochschule
- andere (Aus)Bildung
- Verband/ Fachgesellschaft
- Im Rahmen meiner beruflichen Tätigkeit
- Schule
- Familie
- Freunde
- Vorwiegend eigenständig

**Sind Sie Mitglied in einer Fachgesellschaft für eine bestimmte Artengruppe (z.B. GdO, GAC, DDA etc)?**

Bitte wählen Sie nur eine der folgenden Antworten aus:

- Ja
- Nein

**Wie lauten die ersten zwei Ziffern Ihrer Postleitzahl?**

Bitte geben Sie Ihre Antwort hier ein:

**Haben Sie noch weitere Anmerkungen? Hier können Sie zusätzliche Kommentare zu den Fragen oder zur Umfrage abgeben.**

Bitte geben Sie Ihre Antwort hier ein:

**Vielen Dank für die Teilnahme an dieser Umfrage!**

**Ihre Erfahrung wird uns helfen, bessere Auswertungsmöglichkeiten für Biodiversitätstrends zu entwickeln.**
