# Supplementary material for: Decision-making of citizen scientists when recording species observations
Source: Sci Rep. 2022 Jun 30;12:11069. doi: 10.1038/s41598-022-15218-2 (PMC9245884; doi:10.1038/s41598-022-15218-2)
Supplement: Supplementary file 2 — Supplementary Information 2. [file 41598_2022_15218_MOESM2_ESM.docx]

**sMon Observational Data**

**Survey about the collection of species data**

Dear nature watchers and citizen scientists,

We need your help.

Your committed involvement in the reporting of species observations is very important for biodiversity research. This survey is intended to help us better understand the observation techniques used by nature watchers and citizen scientists in Germany. Your experience will help us to design better analysis methods for biodiversity trends. We are interested in how people decide when to observe species and how to document their observations. Most of these observations occur outside of structured monitoring programs (such as the Butterfly Monitoring Germany – Tagfalter-Monitoring Deutschland). Instead, most observational data are gathered by numerous observers following differing criteria, and reported for instance using apps or online platforms (such as ornitho.de or artenfinder.rlp). In this survey we target this latter kind of observation and its associated methods.

**Who is conducting this survey?**

We are scientists of the German Centre for Integrative Biodiversity Research (iDiv) Halle-Jena-Leipzig and are conducting the survey as part of the project “sMon – Biodiversity trends in Germany” (idiv.de/smon). Within sMon, we collaborate closely with natural history societies, conservation NGOs, government agencies, and research institutes in Germany to evaluate and analyse trends in biodiversity data.

**Who are we trying to reach with this survey?**

This survey is for people who voluntarily collect observations about plants and animals in their free time, and report these observations to an agency or organisation. We encourage people of all experience levels to participate, whether you are a beginner or a sporadic contributor or an experienced observer or even an expert. There are no right or wrong answers for these questions.

**A note on data protection**

Participation in this survey is voluntary. The collection and processing of data collected in this survey conforms to the General Data Protection Regulation (EU-GDPR). The data are only used for scientific research and will never be passed on to third parties. All answers will be anonymised.

**Completing the survey will require approximately 15 minutes.**

If you have any further questions about the content or procedure of the survey, do not hesitate to contact me. There is also space for comments at the end of the survey.

Thank you very much in advance for your participation in this survey.

Dr. Diana Bowler

German Centre for Integrative Biodiversity Research (iDiv) Halle-Jena-Leipzig

Puschstr. 4

04103 Leipzig

E-Mail: [smon@idiv.de](mailto:smon@idiv.de); Web: www.idiv.de/de/smon.html

**Your experiences**

**How many years have you been active in the collection of species observation data?**

Please enter an integer value into this field:

- year(s)

**How often have you collected species data in the spring or summer of 2020?**

Please choose one of the following answers:

- daily / almost daily
- weekly / almost weekly
- every two weeks / almost every two weeks
- monthly / almost monthly
- every two months / almost every two months
- less frequently

**How important were the following aspects to you when you were collecting species observation data?**

Please select the appropriate answer for each item on the list:

|  | not at all important | not very important | of medium importance | important | very important |
| --- | --- | --- | --- | --- | --- |
| Improving my knowledge about the species |  |  |  |  |  |
| Contributing to scientific knowledge |  |  |  |  |  |
| Supporting biodiversity conservation |  |  |  |  |  |
| Spending time outdoors |  |  |  |  |  |
| Physical activity |  |  |  |  |  |
| Protecting/improving nature in a specific place |  |  |  |  |  |
| Gaining local knowledge around home |  |  |  |  |  |
| Meeting other people |  |  |  |  |  |
| Have fun exploring/findings |  |  |  |  |  |

**Which taxonomic group do you observe and report most often?**

**Please choose ONE species group.**

Please select one of the following options:

- Plants
- Beetles
- Dragonflies
- Butterflies
- Bees
- Amphibians/Reptiles
- Birds
- Other (please specify)

**Do you mostly collect observational data about one specific subgroup of this species group (e.g. a family)?**

Please choose one of the following options:

- Yes, I mostly collect data about one specific subgroup.
- No, I do not concentrate on any specific subgroup.
- I don’t know.

**If yes: Please specify the subgroup about which you collect most observational data.**

Please enter your answer here:

**Please answer all following questions with regards to this species group.**

**Which platform(s) do you generally use to submit your species observations?**

Please select all relevant answers:

- naturgucker
- iNaturalist
- Artenfinder
- Naturblick
- I do not use a platform
- Other (please specify):

**Did you observe and report species differently in spring/summer 2020 because of the Covid pandemic, as compared to previous years?**

Please choose one of the following answers:

- No, it was my first year.
- No, I mostly proceeded as in previous years.
- Yes, I was less active than in previous years.
- Yes, I was more active than in previous years.

**In the following questions, we are interested in how you collect species observations.**

**How many of the species observations that you reported in spring/summer 2020 were:**

Please select the appropriate answer for each item on the list:

|  | none | very few | a couple | most | all |
| --- | --- | --- | --- | --- | --- |
| the result of an active search (for example, you drove to a specific location to look for species there |  |  |  |  |  |
| the result of random observations (without an active search) |  |  |  |  |  |
| the result of traps that you set |  |  |  |  |  |

**Active search**

**If you went actively searching for species, how long did you typically search?**

Please enter your answer(s) here:

- in hours
- or in minutes

**If you went actively searching for species, how did you go about collecting observations?**

Please select the appropriate answer for each item on the list:

|  | don’t know | never | rarely | occasionally | often | very often |
| --- | --- | --- | --- | --- | --- | --- |
| I have a checklist with species that can be expected and look through the list. |  |  |  |  |  |  |
| I record all species that I see. |  |  |  |  |  |  |
| I record only the species that I find interesting. |  |  |  |  |  |  |
| I only record common species. |  |  |  |  |  |  |
| I only record rare species. |  |  |  |  |  |  |

**Random observations**

**If you see a species by chance (without actively searching), what prompts you to report the observation? Please state how often the following reasons led you to report an observation.**

Please select the appropriate answer for each item on the list:

|  | don’t know | never | rarely | occasionally | often | very often |
| --- | --- | --- | --- | --- | --- | --- |
| I see a rare species. |  |  |  |  |  |  |
| I see many individuals of the same species. |  |  |  |  |  |  |
| I see many species at the same time. |  |  |  |  |  |  |
| I see a species in a location where I did not expect it. |  |  |  |  |  |  |
| I see a species for the first time this year. |  |  |  |  |  |  |
| I see a species I don‘t know. |  |  |  |  |  |  |
| I see a species I find interesting. |  |  |  |  |  |  |

**Trap setups**

**If you used a trap, how long was the typical recording duration?**

Please enter your answer(s) here:

- in hours
- or in minutes

**What types of traps do you use? (Please separate your answers with a comma.)**

Please enter your answer here:

**What method do you use when recording the species caught in a trap?**

Please select the appropriate answer for each item on the list:

|  | don’t know | never | rarely | occasionally | often | very often |
| --- | --- | --- | --- | --- | --- | --- |
| I record all species. |  |  |  |  |  |  |
| I only record species that I find interesting. |  |  |  |  |  |  |
| I only record common species. |  |  |  |  |  |  |
| I only record rare species. |  |  |  |  |  |  |
| I only record species that are not in my collection yet. |  |  |  |  |  |  |

**Identifying species**

**What do you do if you are uncertain about the identity of a species? Please state, how often you do each of the following:**

Please select the appropriate answer for each item on the list:

|  | don’t know | never | rarely | occasionally | often | very often |
| --- | --- | --- | --- | --- | --- | --- |
| I guess which species it is. |  |  |  |  |  |  |
| I don‘t report the species. |  |  |  |  |  |  |
| I report the lowest taxonomic level I can safely identify (e.g. genus, species). |  |  |  |  |  |  |
| I ask another person to verify my identification. |  |  |  |  |  |  |
| I use relevant resources to help me identify the species (e.g. the Internet or a field guide) |  |  |  |  |  |  |

**Places of observation**

**If you think back to spring/summer 2020, how often did you search for species at the following locations?**

Please select the appropriate answer for each item on the list:

|  | don’t know | never | rarely | occasionally | often | very often |
| --- | --- | --- | --- | --- | --- | --- |
| Protected areas |  |  |  |  |  |  |
| Forest |  |  |  |  |  |  |
| Wetlands or bodies of water |  |  |  |  |  |  |
| Meadows |  |  |  |  |  |  |
| Farmland |  |  |  |  |  |  |
| Urban areas – green spaces (e.g. parks) |  |  |  |  |  |  |
| Urban areas – built-up areas (e.g. houses, pavements, roads) |  |  |  |  |  |  |
| Remote areas (e.g. more than 50 km from the nearest town) |  |  |  |  |  |  |

**Imagine you visit a place that you‘ve been to before. There, you see a species that you already saw and reported on your last visit. How likely is it that you would report this new sighting, if the last visit was… ?**

Please select the appropriate answer for each item on the list:

|  | don’t know | very unlikely | unlikely | somewhat likely | likely | very likely |
| --- | --- | --- | --- | --- | --- | --- |
| … on the same day. |  |  |  |  |  |  |
| … in the same week. |  |  |  |  |  |  |
| … in the same month. |  |  |  |  |  |  |
| … in the same year. |  |  |  |  |  |  |
| …in a previous year. |  |  |  |  |  |  |

**Personal details**

Finally, we would like to collect some data about yourself. You will remain anonymous – the answers to these questions will not enable anybody to identify any survey participants. If you do not want to give any personal details, click “Submit” below.

**I am**

Please choose one of the following options:

- female
- male
- other

**To which age group do you belong?**

Please choose one of the following options:

- 19 or younger
- 20-29
- 30-39
- 40-49
- 50-59
- 60-69
- 70-79
- 80+

**Do you participate in a large-scale standardised monitoring scheme (e.g. Butterfly Monitoring Deutschland – Tagfalter Monitoring Deutschland)?**

Please choose one of the following options:

- Yes
- No

**Do you have expertise in the area of biodiversity monitoring?**

Please choose one of the following options:

- Yes
- No

**Where did you mainly gain this expertise?**

Please choose one of the following options:

- university / college
- other education / vocational training
- natural history society / conservation NGO
- through my professional work
- school
- family
- friends
- mostly self-taught

**Are you a member of a natural history society for a specific taxon group (e.g. GdO, GAC, DDA, etc.)?**

Please choose one of the following options:

- Yes
- No

**What are the first two figures of your ZIP code?**

Please enter your answer here:

**Do you have any further comments? Here you can enter additional comments about the survey.**

Please enter your answer here:

**Thank you very much for participating in this survey! Your experience will help us design better analysis methods for biodiversity trends.**
