# Supplementary material for: Decision-making of citizen scientists when recording species observations
Source: Sci Rep. 2022 Jun 30;12:11069. doi: 10.1038/s41598-022-15218-2 (PMC9245884; doi:10.1038/s41598-022-15218-2)
Supplement: Supplementary file 3 — Supplementary Information 3. [file 41598_2022_15218_MOESM3_ESM.docx]

Supplementary material for:

Decision-making of citizen scientists when recording species observations

Diana E. Bowler, Netra Bhandari, Lydia Repke, Christoph Beuthner, Corey T. Callaghan, David Eichenberg, Klaus Henle, Reinhard Klenke, Anett Richter, Florian Jansen, Helge Bruelheide, Aletta Bonn

Corresponding author: [diana.e.bowler@gmail.com](mailto:diana.e.bowler@gmail.com)

Contents:

Table S1-S2

Figures S1-S8

Table S1 Component items of the main questions analysed in the paper

| Question group | Component items |
| --- | --- |
| Experience  - of biodiversity monitoring | number of years collecting data  frequency of collecting data  participation in a large-scale monitoring scheme  formal knowledge/expertise  member of a natural history society |
| Motivations  - for collecting species data | improve knowledge of species  support conservation  have fun exploring/finding  contribute to scientific knowledge  spend time outdoors  protect/maintain/improve a specific place  gain local knowledge about where I live  physical activity  meet other people |
| survey types  - what proportion of data come from different survey types? | active and planned searches  opportunistic observations  using traps |
| active searches  - how often are different species reported? | all species seen  interesting species  rare species  common species  expected species on a checklist |
| opportunistic observations  - what triggers recording an opportunistically observed species? | rare species  interesting species  first time to see a species in a year  species in an unexpected location  many individuals of a species  many species at the same time  unknown species |
| species ID uncertainty  - how did you deal with taxonomic ID uncertainty? | use identification aids e.g. guidebooks  guess the species ID  report the observation at a higher taxonomic level  not report the species  ask another person to check |
| locations  - how often did you search for species in the following locations? | protected areas  forest  meadow  farmland  urban areas (green e.g., parks)  urban areas (built e.g., houses, roads)  remote areas (>50 km from town) |

Table S2 Example of traps reported to be used for different taxonomic groups

| Taxa | Types of traps used |
| --- | --- |
| Butterflies/Moths | light trap, pheromone trap, wine-sugar-baited trap, Quartz lamp, superactinic fluorescent tube, Mixed light lamp 250 Watt, Light trap 15W superactinic, funnel trap, red wine bait, Robinson trap, paint tray, malaise trap, bait trap, bait paint, funnel trap with light. |
| Amphibians/Reptiles | box trap with 4 illuminated openings, coverboards, drift fences with pitfall or bucket traps, bottle trap, bucket traps, funnel traps, plastic funnel traps with lights (for newts), water traps, fish traps (reusen), nets/netting, Orthmann funnel trap, minnow traps, hemp trap, traps from gulls and channels |
| Beetles | pitfall traps, window traps, funnel traps, light traps, pheromone traps, glue traps, Barber pitfall traps, photo-eclector traps, baited trap, aerial funnel trap, carnet, Malaise traps |
| Other | light traps, Malaise traps, coloured pan traps, emergence traps, acoustic recorders, fishing lines, baskets, camera traps, real-time ultrasound recorder, nets, tubes, sticky traps, Barber pitfall traps, dormouse box, live mousetraps, fish traps |


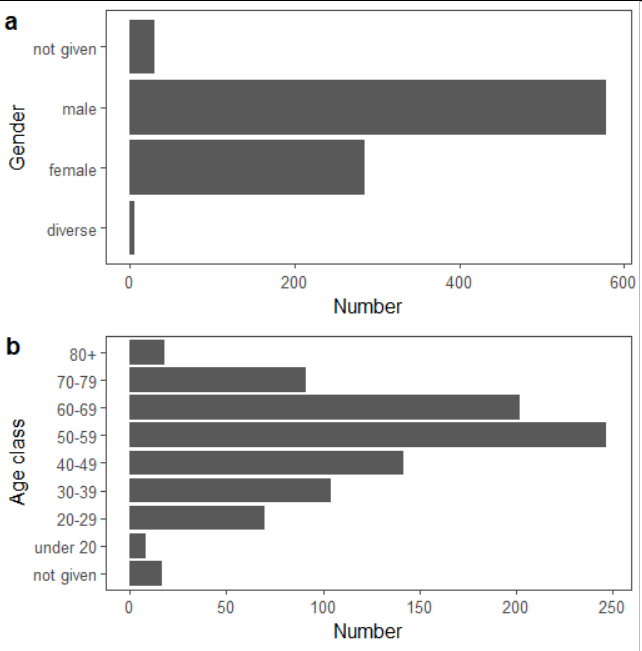


Fig. S1 Demographics of respondents taking part in our survey, split by (a) gender and (b) age (total n = 899).


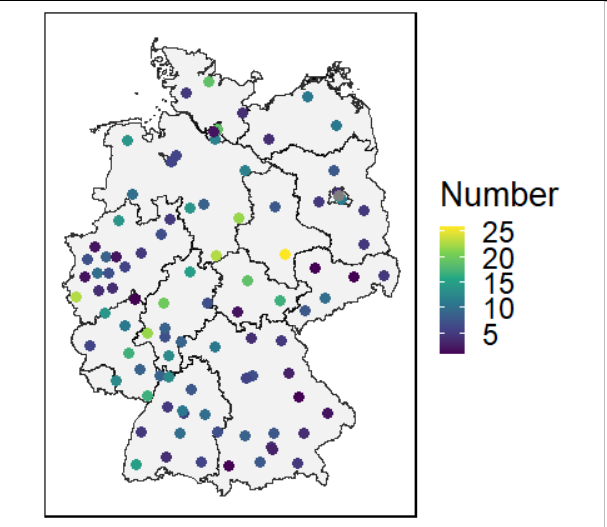


Fig. S2 Map showing the number of respondents per postcode location of their home address (first 2 letters of postcode only). Colours reflect the number of respondents of the same postcode. Federal state borders are also shown.


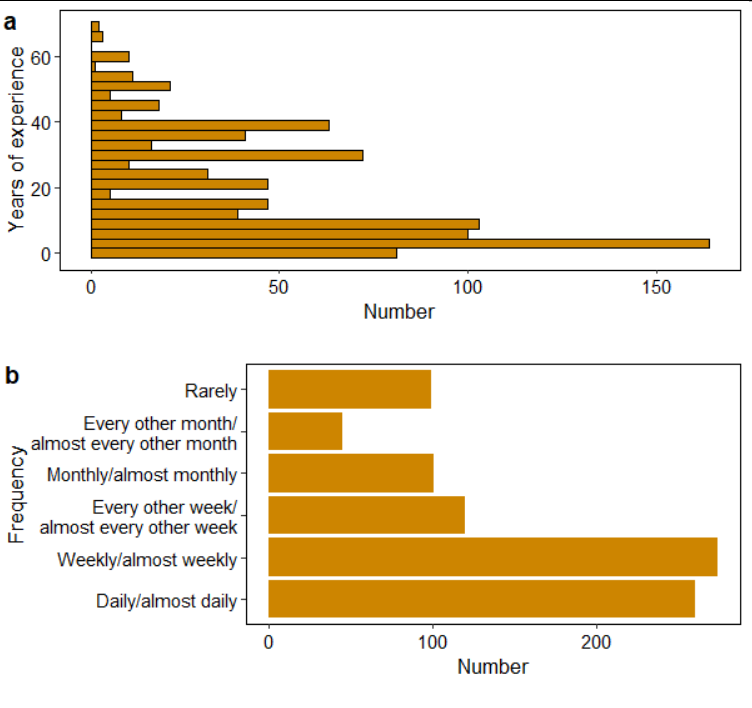


Fig. S3 Demographics of respondents taking part in our questionnaire, split by (a) number of years of experience and (b) frequency of collecting species observation during Spring or Summer 2020.


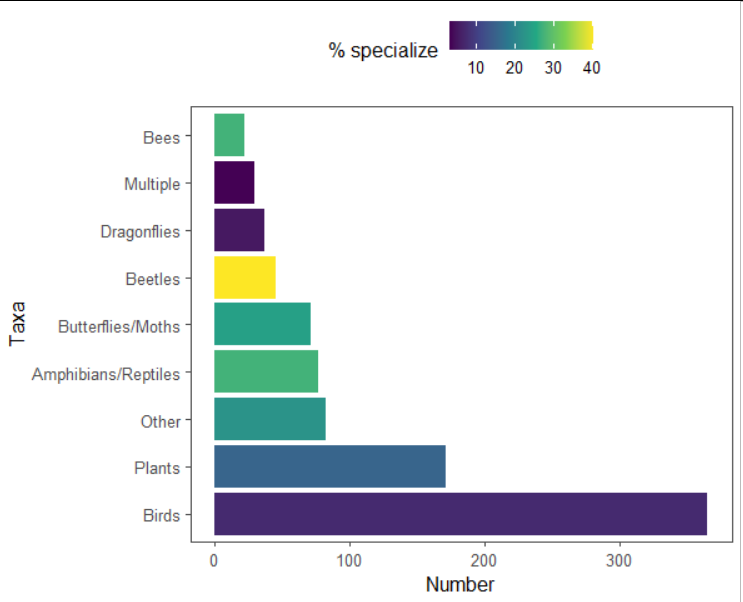


Fig. S4 Number of respondents named each taxa group as their focal group. Colours reflect the percentage of people who specialized on a subset of taxon within the group. Specialism was highest within beetles and lowest within birds and dragonflies.


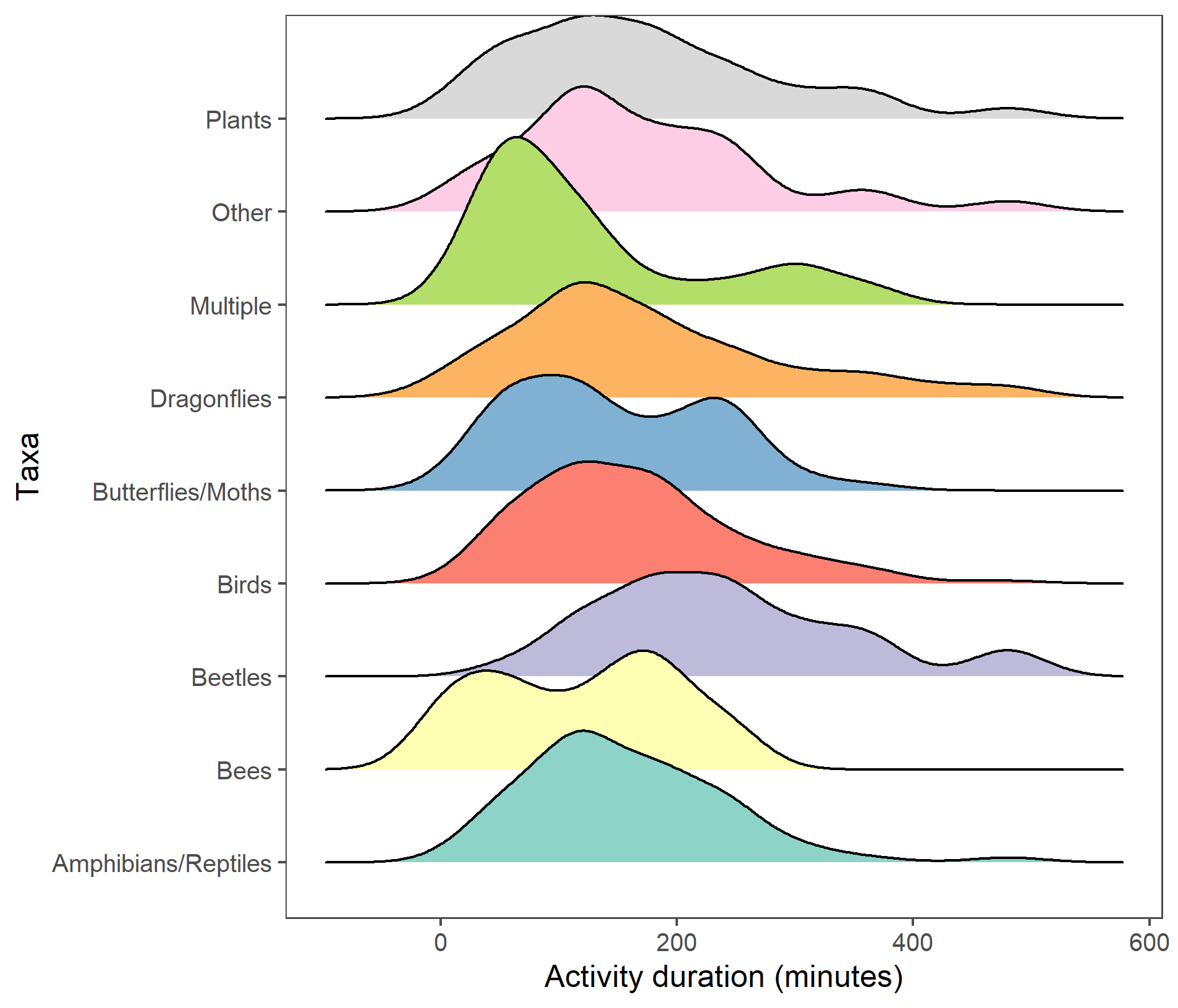


Figure S5 Distribution of survey effort (activity duration) during active searches for the different taxa.


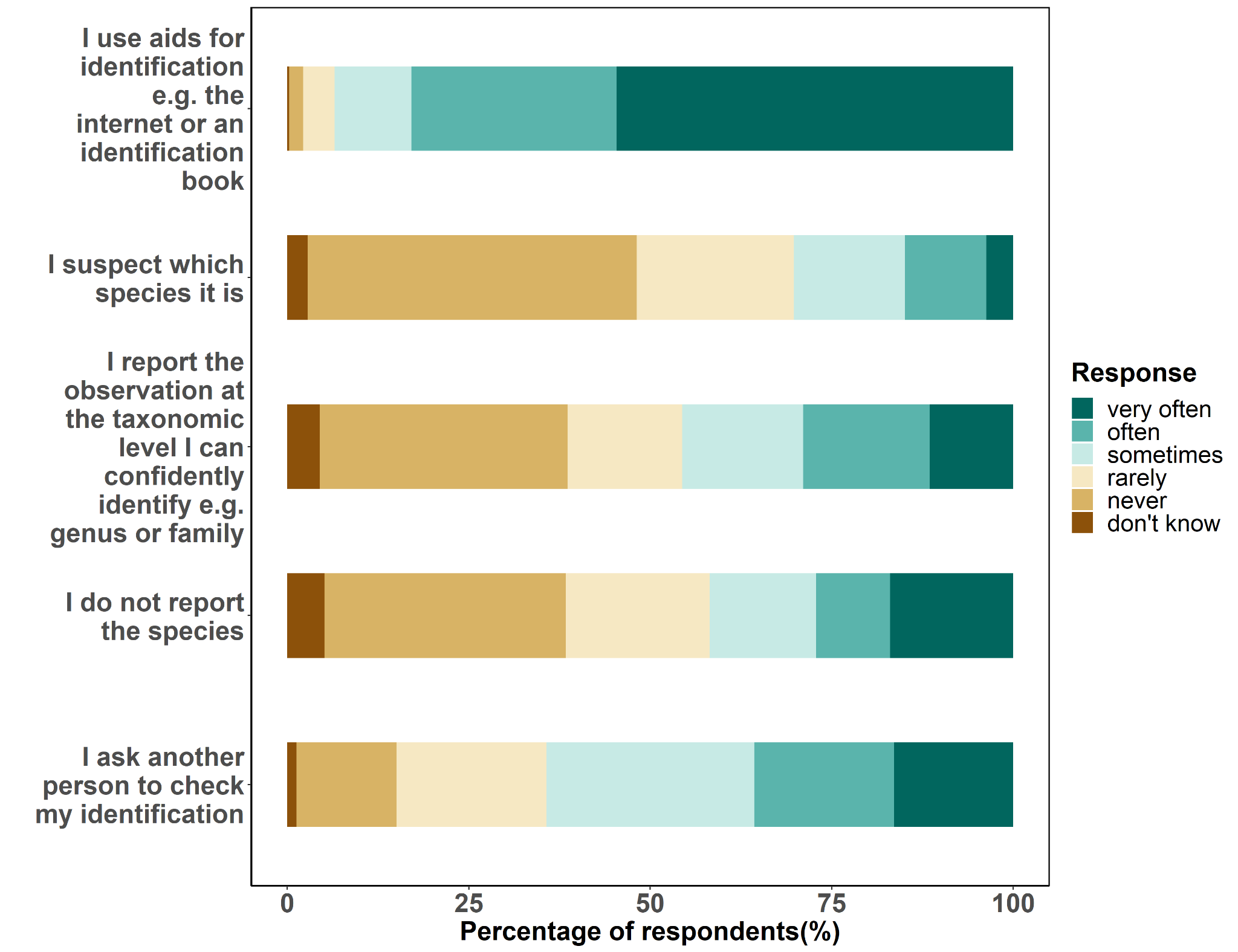


Fig. S6 Approaches for dealing with species identification uncertainty.


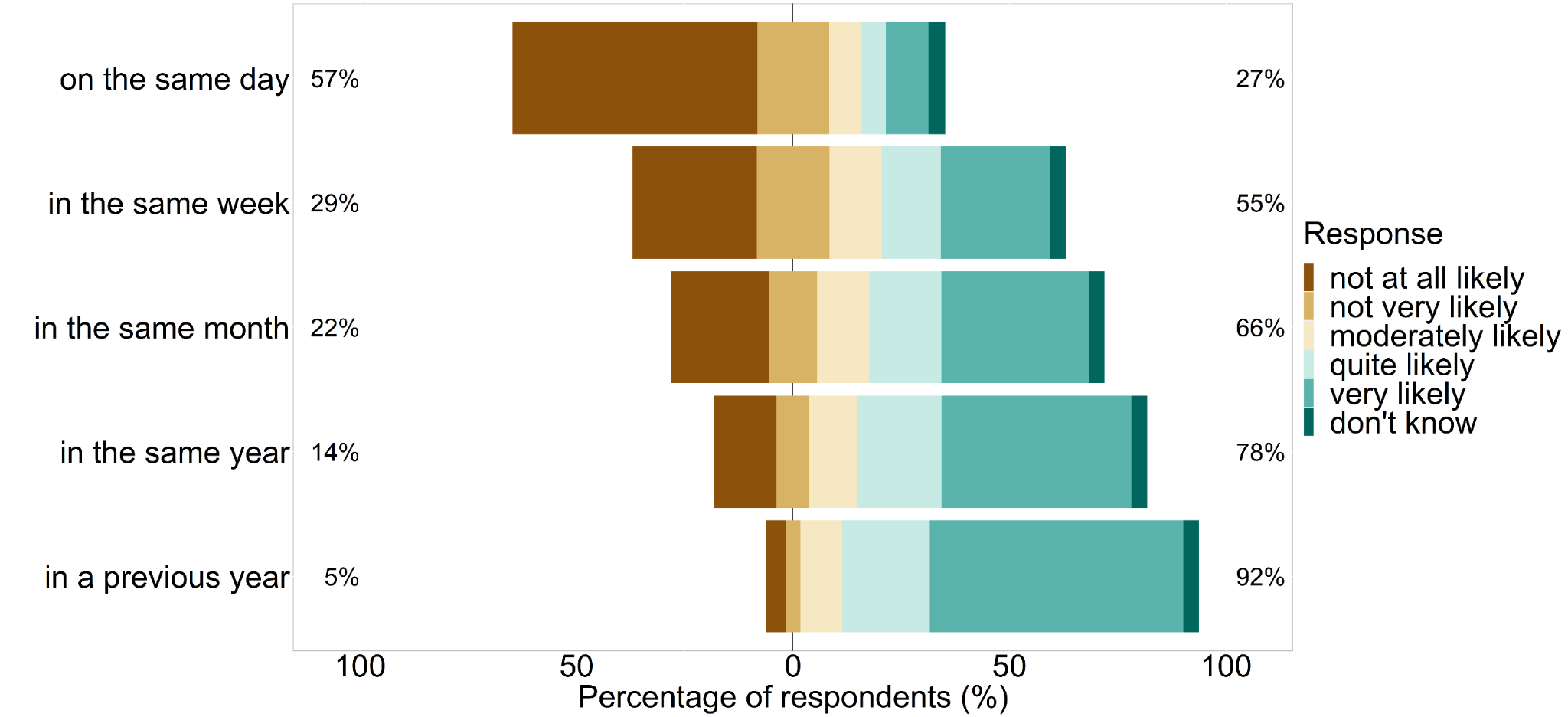


Figure S7 Consecutive surveys: likelihood of reporting a species observation when the same species was already reported from the same place in different previous time points.


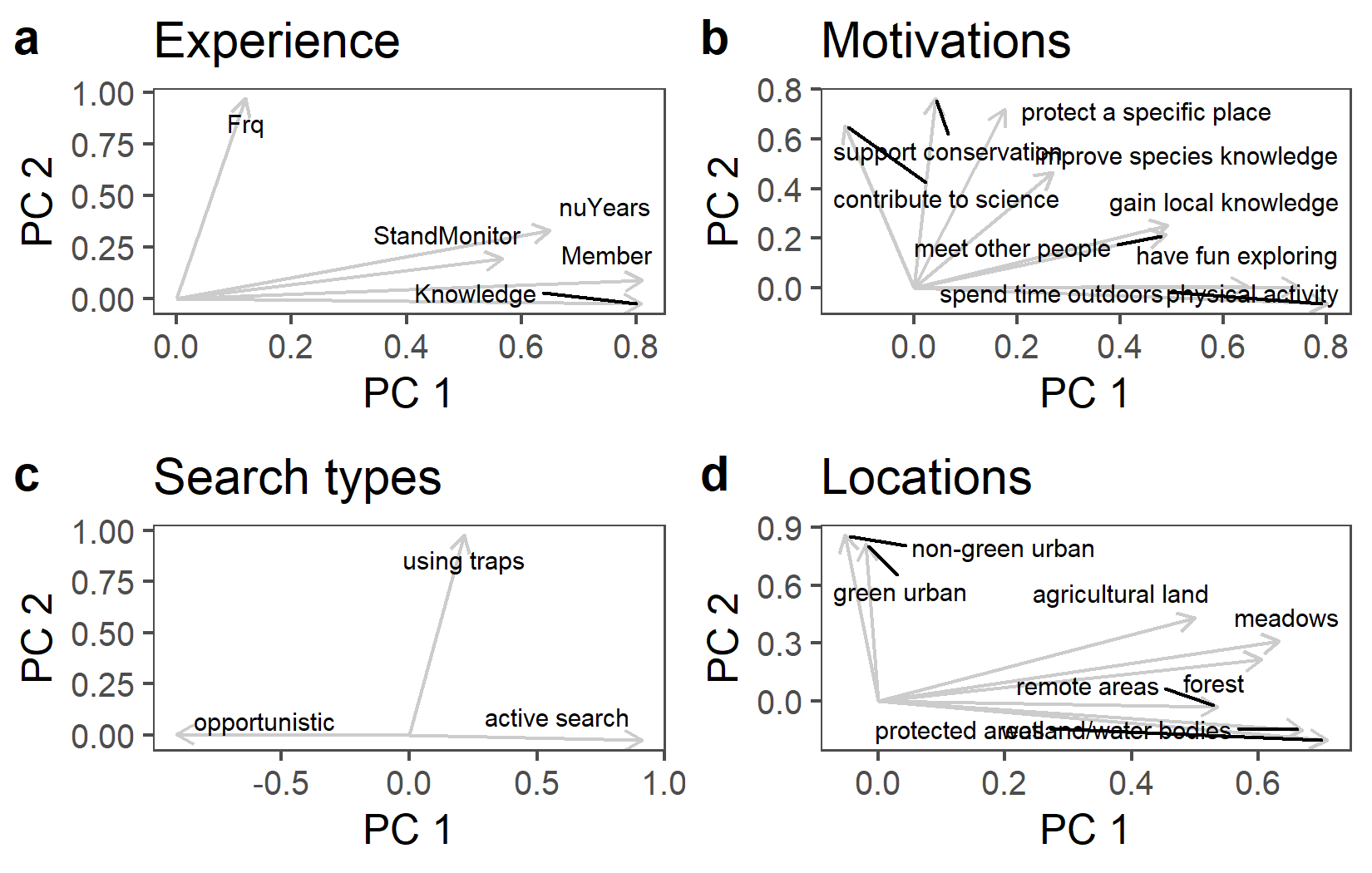


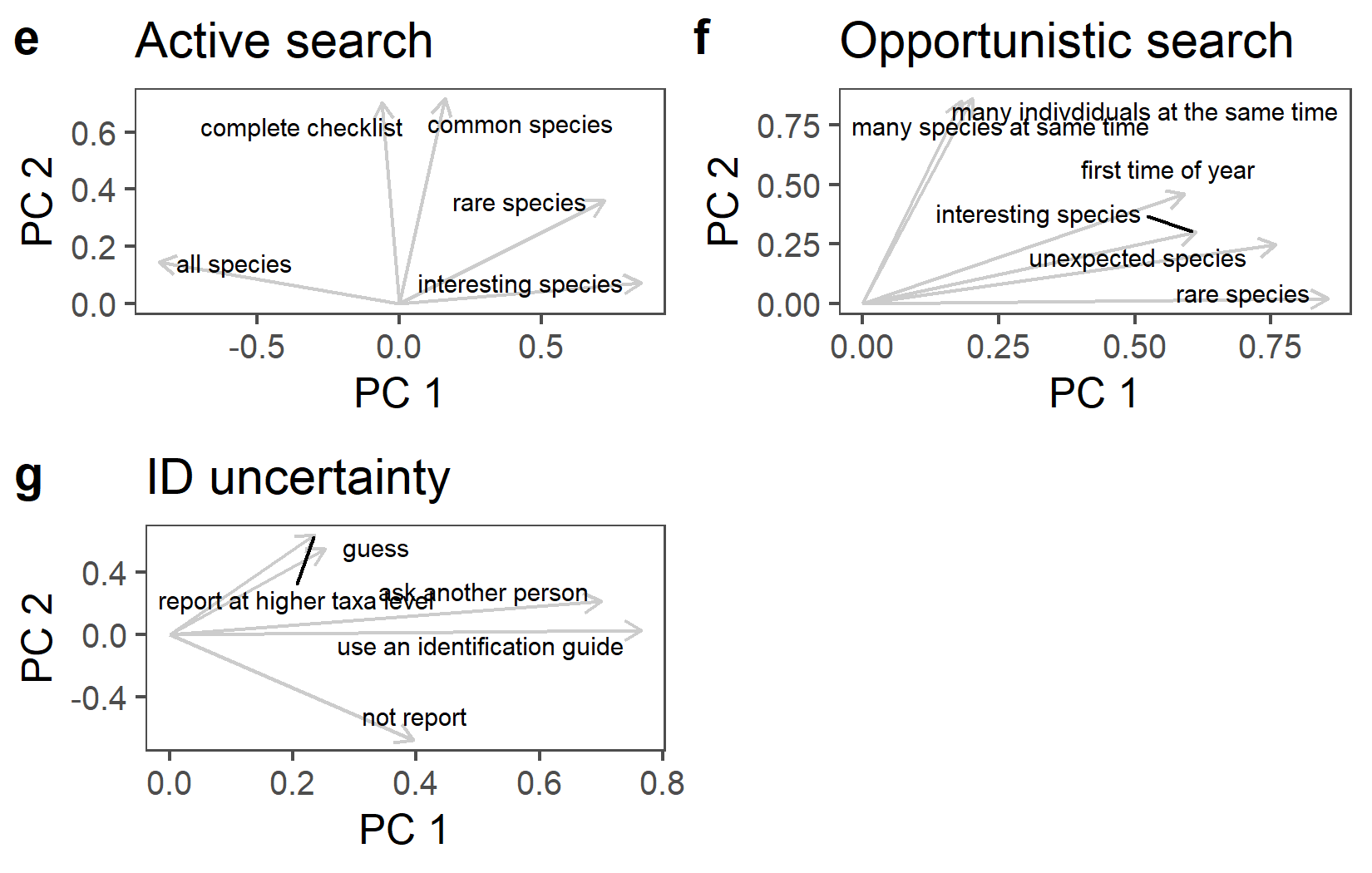


Fig. S8 Biplots of the PCAs of items within each question.
